# An Optimized Mouse Brain Atlas for Automated Mapping and Quantification of Neuronal Activity Using iDISCO+ and Light Sheet Fluorescence Microscopy

**DOI:** 10.1007/s12021-020-09490-8

**Published:** 2020-10-16

**Authors:** Johanna Perens, Casper Gravesen Salinas, Jacob Lercke Skytte, Urmas Roostalu, Anders Bjorholm Dahl, Tim B. Dyrby, Franziska Wichern, Pernille Barkholt, Niels Vrang, Jacob Jelsing, Jacob Hecksher-Sørensen

**Affiliations:** 1 Gubra ApS, 2970 Hørsholm, Denmark; 2 Department of Applied Mathematics and Computer Science, Technical University Denmark, 2800 Kongens Lyngby, Denmark; 3 Danish Research Centre for Magnetic Resonance, Centre for Functional and Diagnostic Imaging and Research, Copenhagen University Hospital Hvidovre, 2650 Hvidovre, Denmark

**Keywords:** Light sheet fluorescence microscopy, iDISCO, Tissue clearing, Brain atlas, C-Fos, Whole brain imaging

## Abstract

In recent years, the combination of whole-brain immunolabelling, light sheet fluorescence microscopy (LSFM) and subsequent registration of data with a common reference atlas, has enabled 3D visualization and quantification of fluorescent markers or tracers in the adult mouse brain. Today, the common coordinate framework version 3 developed by the Allen’s Institute of Brain Science (AIBS CCFv3), is widely used as the standard brain atlas for registration of LSFM data. However, the AIBS CCFv3 is based on histological processing and imaging modalities different from those used for LSFM imaging and consequently, the data differ in both tissue contrast and morphology. To improve the accuracy and speed by which LSFM-imaged whole-brain data can be registered and quantified, we have created an optimized digital mouse brain atlas based on immunolabelled and solvent-cleared brains. Compared to the AIBS CCFv3 atlas, our atlas resulted in faster and more accurate mapping of neuronal activity as measured by c-Fos expression, especially in the hindbrain. We further demonstrated utility of the LSFM atlas by comparing whole-brain quantitative changes in c-Fos expression following acute administration of semaglutide in lean and diet-induced obese mice. In combination with an improved algorithm for c-Fos detection, the LSFM atlas enables unbiased and computationally efficient characterization of drug effects on whole-brain neuronal activity patterns. In conclusion, we established an optimized reference atlas for more precise mapping of fluorescent markers, including c-Fos, in mouse brains processed for LSFM.

**Electronic supplementary material:**

The online version of this article (10.1007/s12021-020-09490-8) contains supplementary material, which is available to authorized users.

## Introduction

Rodent models are important tools in preclinical drug development for central nervous system (CNS) disorders (Bobela et al. 2014; Esquerda-Canals et al. 2017; Leung and Jia 2016). A common method for characterizing central effects of potential novel therapies is to quantify expression patterns of c-Fos, a proxy for neuronal activation (Dragunow and Faull 1989).

Recent advances in immunohistochemical methods and optical clearing techniques have, together with ex vivo imaging technologies such as light sheet fluorescence microscopy (LSFM), enabled whole-organ imaging (Chung et al. 2013; Ertürk et al. 2012; Jensen et al. 2015; Kjaergaard et al. 2019; Renier et al. 2014; Rocha et al. 2019; Secher et al. 2014). As a result, it is now possible to visualize c-Fos expression at the single cell level in the intact adult mouse brain (Kjaergaard et al. 2019; Nectow et al. 2017; Renier et al. 2016).

In recent years, automated image analysis algorithms have been developed enabling 3D quantification of activated neurons and their signal intensities in the adult mouse brain (Detrez et al. 2019; Jensen et al. 2015; Liebmann et al. 2016; Nectow et al. 2017; Salinas et al. 2018; Schneeberger et al. 2019). The first step of the analysis process is to register LSFM imaging data onto a common reference brain which contains annotated brain regions. Today, the most widely used mouse brain atlas is the common coordinate framework version 3 (CCFv3), developed by the Allen Institute for Brain Science (AIBS) (Allen Institute for Brain Science 2011, 2015, 2017; Kuan et al. 2015; Wang et al. 2020). For quantification of fluorescent signals, registration is followed by cell detection, e.g. ClearMap, to segment and count c-Fos positive cells (Nectow et al. 2017; Renier et al. 2016) or extract voxel intensities (Salinas et al. 2018). Finally, the results can be assigned to specific regions using the anatomical reference atlases such as those provided by AIBS.

LSFM image processing pipelines have improved quantitative whole-brain 3D imaging. However, the quality of the LSFM results is highly dependent on sample processing and the imaging methods applied. Whole-organ immunolabelling requires lipid extraction to make the tissue permeable to antibodies (Kim et al. 2018) and enable deep tissue imaging (Vigouroux et al. 2017). In particular, myelin fibers which are lipid-rich (Villares et al. 2015), are more likely to be affected by lipid extraction, leading to non-uniform morphological changes within the brain. Also, various clearing medias have different chemical properties which will result in either shrinkage or expansion of brain structures (Wan et al. 2018). In contrast, the AIBS CCFv3 is based on vibratome-sectioned and two-photon microscopy imaged brains. Consequently, brains imaged with LSFM differ from the AIBS CCFv3 atlas with respect to morphology and signal intensity. This affects the registration accuracy and because the morphological changes introduced by the sample processing are tissue-dependent, some brain regions are more prone to erroneous alignment than others. As result, subsequent data analysis requires time-consuming validation and manual correction to ensure accurate quantification. This is particularly relevant in pre-clinical research where group sizes are often relatively large in order to provide better statistical power.

In our experience, the hindbrain is particularly sensitive to erroneous registration when cleared samples are mapped directly onto the AIBS CCFv3. High quality registration can be achieved using a multi-regional approach where each larger part of the brain, e.g. the hindbrain, is registered separately. However, this procedure reduces analysis speed as initial segmentation of the larger brain structures is required. We aimed to preserve both data flow and quality by generating a reference template based on iDISCO+ processed and LSFM-imaged mouse brains and aligning the AIBS CCFv3 with the template through multi-regional registration.

The LSFM atlas enables fast brain-wide inter-modality registration of other LSFM samples. To confirm accuracy and demonstrate the utility of the LSFM-based reference brain atlas, we determined the c-Fos expression signature of semaglutide, a long-acting glucagon-like peptide-1 (GLP-1) receptor agonist. The LSFM atlas enabled precise mapping of semaglutide-induced c-Fos expression in the mouse whole-brain. In addition to c-Fos imaging, application of the atlas includes also mapping other fluorescent markers imaged by LSFM.

## Materials and Methods

### Animals

Male C57Bl/6 J mice were obtained from Janvier Labs (Le Genest-Saint-Isle, France), and were maintained in standard housing conditions (12 h light/dark cycle and controlled temperature of 21–23 °C). Mice had ad libitum access to tap water and regular chow (Altromin 1324, Brogaarden, Hørsholm, Denmark) or high fat diet (60% fat, 21% carbohydrates, 19% protein; Ssniff Spezialdiäten GmbH, Soest, Germany). The LSFM atlas was established based on analysis of 139 brains from 8 to 10 weeks old male chow-fed mice. The pharmacology-induced neuronal activity study involved two groups of lean mice and two groups of DIO mice. All groups were aged matched (38 weeks) and consisted of *n* = 6. Lean and DIO control group animals received phosphate buffered saline with BSA, lean and DIO treatment group animals received semaglutide (Ozempic®, Novo Nordisk A/S, Bagsværd, Denmark) dose of 0.04 mg/kg. Both groups were administered subcutaneously 5 ml/kg and the animals were sacrificed 4 h post-dose. All animal procedures were conducted in compliance with internationally accepted principles for the care and use of laboratory animals and were approved by the Danish Animal Experiments Inspectorate (license #2013-15-2934-00784).

### Sample Preparation for Immunohistochemistry

Animals were transcardially perfused with heparinized PBS and 40 ml of 10% neutral buffered formalin (CellPath, Newtown, UK) under Hypnorm-Dormicum (fentanyl 788 μg/kg, fluanisone 25 mg/kg and midazolam 12.5 mg/kg, subcutaneous injection) anesthesia. Brains were carefully dissected and immersion-fixed in 10% neutral buffered formalin overnight at room temperature on a horizontal shaker. Whole-brain samples were washed 3 × 30 min in PBS with shaking and dehydrated at room temperature in methanol/H_2_O gradient to 100% methanol (20%, 40%, 60%, 80%, 100% methanol, each step 1 h). The brains were stored in 100% methanol (VWR International A/S, Søborg, Denmark) at 4 °C until further processing.

### Whole-Brain Immunohistochemistry for Labeling of c-Fos Positive Cells and Clearing

The iDISCO+ (immunolabeling-enabled three-dimensional imaging of solvent-cleared organs) protocol was used for whole brain immunolabelling (Renier et al. 2014, 2016). Samples were washed with 100% methanol for 1 h and incubated overnight in 66% dichloromethane/33% methanol (VWR International A/S, Søborg, Denmark) at room temperature. Then, samples were washed twice in 100% methanol for 30 min and bleached in chilled fresh 5% H_2_O_2_ (Acros Organics, Fisher Scientific Biotech Line A/S, Slangerup, Denmark) in methanol overnight at 4 °C. Subsequently, the samples were rehydrated in methanol/PBS series (80%, 60%, 40%, 20% methanol with 0.2% Triton X-100 (Merck, Darmstadt, Germany), each step 1 h) at room temperature, washed in PBS with 0.2% Triton X-100 twice for 1 h at room temperature and in permeabilization solution (PBS with 0.2% Triton X-100, supplemented with 20% volume of DMSO (Merck, Darmstadt, Germany) and 2.3% weight/volume glycine (Merck, Darmstact, Germany)) for 3 days at 37 °C. For c-Fos labeling, unspecific antibody binding was blocked in blocking solution (PBS, 0.2% TritonX-100, 10% DMSO/6% donkey serum (Jackson ImmunoResearch, Cambridgeshire, UK)) for 2 days at 37 °C before incubated in the primary antibody buffer (PTwH, 5% DMSO, 3% donkey serum, 0.2% of 10% NaN_3_ (Merck, Darmstadt, Germany)) for 7 days at 37 °C. For visualization of c-Fos expression, rabbit anti-c-Fos antibody (1:5000, Cell Signaling Technology, Massachusetts, US, cat number #2250) was used. Following incubation with primary antibody, the brains were washed in PTwH (PBS, 0.2% Tween 20 (Merck, Darmstadt, Germany), 0.1% of 10 mg/ml heparin solution) for 1 × 10 min, 1 × 20 min, 1 × 30 min, 1 × 1 h, 1× 2 h and 1× 2 days. Subsequently, the brains were incubated in secondary antibody solution (PTwH, 3% donkey serum, 0.2% of 10% NaN_3_) for 7 days at 37 °C with donkey anti rabbit Cy-5 antibody (1:1000, Jackson ImmunoResearch, Cambridgeshire, UK, cat no #711–175-152) and washed in PTwH for 1 × 10 min, 1 × 20 min, 1 × 30 min, 1 × 1 h, 1× 2 h and 1× 3 days. For clearing, the brains were dehydrated in a methanol/H2O series (20%, 40%, 60%, 80% and 100% methanol, each step 1 h) at room temperature, incubated in 66% dichloromethane/33% methanol for 3 h at room temperature with shaking and in 100% dichloromethane twice for 15 min with shaking to remove traces of methanol. Finally, the samples were transferred to dibenzyl ether (Merck, Darmstadt, Germany) and stored in closed glass vials until imaged with light sheet fluorescence microscope.

### Light Sheet Fluorescence Microscopy of Labeled and Cleared Mouse Brains

All whole-brain samples were imaged in an axial orientation on a LaVision ultramicroscope II setup (Miltenyi Biotec, Bergisch Gladbach, Germany) equipped with a Zyla 4.2P-CL10 sCMOS camera (Andor Technology, Belfast, UK), SuperK EXTREME supercontinuum white-light laser EXR-15 (NKT photonics, Birkerød, Denmark) and MV PLAPO 2XC (Olympus, Tokyo, Japan) objective lens. The samples were attached to the sample holder with neutral silicone and imaged in a chamber filled with dibenzyl ether. Version 7 of the Imspector microscope controller software was used. Images were acquired at 0.63 x magnification (1.2 × total magnification) with an exposure time of 254.47 ms in a z-stack at 10 μm intervals. Acquired volumes (16-bit tiff) had an in-plane resolution of 4.8 μm and z-resolution of 3.78 μm (NA = 0.156). Horizontal focusing was captured in 9 planes with blending mode set to the centre of the image to merge the individual raw images. Data was acquired in two channels, autofluorescence and antibody-specific channel, because the former provides information on tissue structure and the latter on neuronal activity. Autofluorescence volumes were acquired at excitation wavelength of 560 ± 20 nm and emission wavelength of 650 ± 25 nm, laser power was set to 80%. Fluorescently labelled c-Fos positive cells were captured in a specific channel at excitation wavelength of 630 ± 15 nm and emission wavelength of 680 ± 15 nm, laser power was set to 100%.

### Image Processing for Creating the Mouse Brain Atlas

**Fig. 1:**
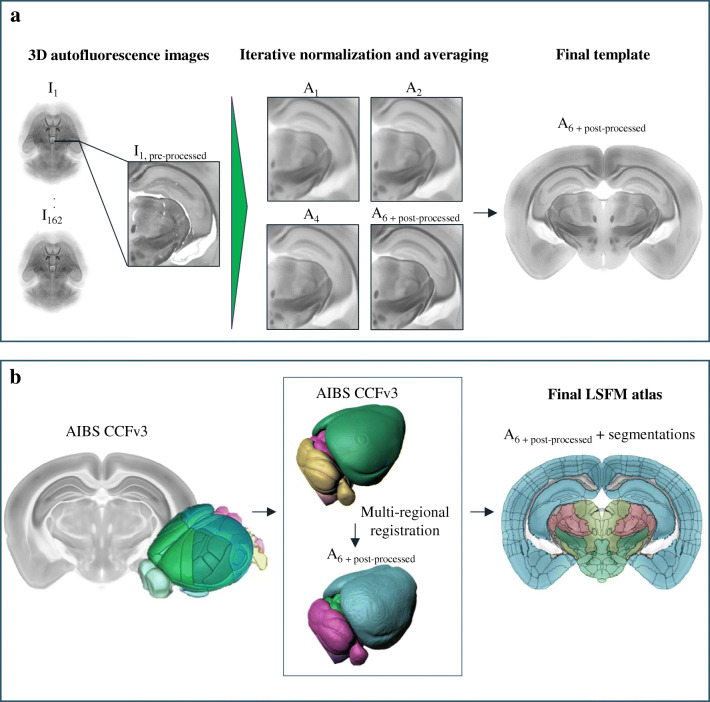
**LSFM-based mouse brain atlas.** a) Generation of a brain template based on the LSFM autofluorescence volumes of 139 mice brains using an iterative registration and averaging algorithm. Raw light sheet scans are annotated with I_x_ where x stands for the animal number, and the intermediate average mouse brain volumes are annotated with A_y_ where y stands for the iteration step. B) Transfer of brain region segmentations from the AIBS CCFv3 to the LSFM mouse brain template. Brain regions of the AIBS CCFv3 were mapped to the LSFM template in six parts, e.g. cortex to cortex, hindbrain to hindbrain etc.

An average LSFM mouse brain volume was created from 139 individual mouse brain autofluorescence datasets by an iterative multi-resolution image registration algorithm (Kovačević et al. 2005; Kuan et al. 2015; Umadevi Venkataraju et al. 2019). Pre-processing was initiated by down-sampling to 20 μm isotropic resolution. N3 method (Larsen et al. 2014; Sled et al. 1998; Van Leemput et al. 1999) was applied to correct intensity inhomogeneity. Subsequently, the intensity histograms of the individual volumes were normalized and, contrast adaptive histogram equalization was performed (Fig. 1a, left). For generating an average mouse brain template, a reference volume was randomly selected as a starting point. Six iterative multi-resolution registration steps – one affine and five B-spline transformations were performed for the remaining samples (Fig. 1a, middle). In the first step the brains were registered to the chosen reference brain and in subsequent steps aligned to the average of all brains from the previous step.

 Due to the limit in scanning depth in the Z-dimension, which is about 6 mm for our LSFM setup, about half a millimetre of the dorsal cortex was not imaged. To produce a template with full cortex, 15 additional image stacks of cortices were acquired, pre-processed and aligned to the average mouse brain volume. Subsequently, both volumes were merged. Satisfactory axial symmetry was achieved by dividing the template brain volume into three coronal slabs with equal thickness and manually rotating them into correct position. The final template was created by mirroring one hemisphere to the opposite side and merging the hemispheres with a sigmoidal blending function for receiving a symmetric template brain (Fig. 1a, right) Additionally, a tissue mask and a ventricular mask were added to the LSFM template from the AIBS CCFv3 and manually adapted to fit the template. 

Brain regional annotations were transferred to the LSFM template from the AIBS CCFv3 (Fig. 1b) (Allen Institute for Brain Science 2011, 2015, 2017; Kuan et al. 2015; Wang et al. 2020). First, the mouse brain template of AIBS was registered onto the LSFM template using multi-resolution affine and B-spline registration. Subsequently, the registered AIBS CCFv3 template and its segmentations were divided into six parental brain regions – cerebral cortex, cerebral nuclei, hindbrain, cerebellum, septal regions and interbrain together with midbrain. The parental regions were then separately registered to the corresponding areas of the LSFM template. Manual corrections were performed for regions near to ventricular system, such as AP and SFO. Segmentation refinements were performed with microscopy image analysis software Imaris™ version 2 (Oxford instruments, Abington, UK). Image processing was performed in Python and Elastix toolbox (Klein et al. 2010; Shamonin et al. 2014) was used to implement the registrations. Detailed description of the atlas creation procedure and full sets of parameters can be found in the Online Resource 1.

### Quantification of c-Fos Positive Cells

Neuronal activity was quantified by detecting and counting c-Fos positive cells using an adapted ClearMap routine (Renier et al. 2016). In brief, the volume pairs collected from the autofluorescence and c-Fos specific channel were aligned slice-by-slice using affine registration in 2D with mattes mutual information as a similarity measure and background subtracted through morphological opening using a disk element. For removing false positive c-Fos signal originating from increased tissue autofluorescence, a signal appearing both in the autofluorescence and the c-Fos specific channel was removed from the specific channel. For identifying c-Fos positive cells, local intensity peaks were monitored by moving a filter cube over the specific channel volume followed by seeded watershed for segmenting the c-Fos positive cells. The initial parameters were taken from the original ClearMap implementation (Renier et al. 2016) but optimized to fit our data, being acquired under different conditions, including image resolution. The size of the filter cube was set to 5x5x3 pixels for effectively detecting all possible c-Fos positive cell candidates. The third dimension of the filter cube was chosen to be smaller than the first and second dimension of the cube since z-resolution of the LSFM volumes was lower than the in-plane resolution. The coordinates of the detected local intensity peaks were used as seeds in watershed segmentation with a background intensity cut-off of 800 and the resulting segmentations were filtered by removing cell segmentation regions smaller than 8 voxels and bigger than 194 voxels. Following c-Fos positive cell detection in the specific channel, the corresponding autofluorescence volumes underwent bias field correction and contrast limited adaptive histogram equalization (similar procedure as for the LSFM mouse brain template creation). For quantifying the number of c-Fos positive cells in individual brain regions, the LSFM atlas was aligned to c-Fos specific channel volumes of individual mice over pre-processed autofluorescence volumes and the number of c-Fos positive was counted in every brain region. Heatmaps visualizing the density of the c-Fos positive cells were created by mapping the specific channel volumes to the LSFM atlas using the inverse transform, generating and summing the spheres of uniform value and 20 μm radius around the centers of the c-Fos positive cells (Renier et al. 2016). Image processing and analysis was performed in Python. 3D visualizations of heatmaps were created with microscopy image analysis software Imaris™ version 2 (Oxford instruments, Abington, UK).

### Statistics

For simplicity, 666 individual brain region segmentations of the LSFM atlas were collapsed to their parental regions using the hierarchy tree of the atlas ontology (Online Resource 2) resulting in 284 regions in which the statistical analysis was performed. For determining the difference in the c-Fos positive cell counts, a generalized linear model was fitted to the cell counts observed in each brain region in every animal group. A negative binomial generalized linear model provided a suitable fit to our c-Fos cell count data. For each generalized linear model, a Dunnett’s test was performed. Statistical analysis for determining differences in c-Fos expression between semaglutide and vehicle treated mice involved *p* value adjustment using a multiple comparison method called false discovery rate. Statistical analysis of the data was performed using R statistics library. 

Further, all significantly regulated brain regions underwent a two-step manual validation procedure for checking if the used statistical model fits the data points, the significance of the brain regions is not achieved due to outliers and the raw signal is truly originating from the region. First, the fit of cell counts to the generalized linear model was evaluated. This was done by investigating deviance residuals and checking if the residuals aligned with the assumptions of normality and homoscedasticity. Furthermore, Cook’s distance was calculated for each cell count data point in the model as a measure of model influence. Regions where the generalized linear model showed severe violations of the assumptions, or the model contained overly influential data points, were discarded. Secondly, the remaining brain regions were visually studied for possible spillover signal from neighboring regions. If the c-Fos response in a region seemed to originate from the neighboring region, e.g. very few c-Fos positive cells were observed only in the border areas of the region while the neighboring areas were exhibiting very high signal, it was declared as not significant.

## Results

### LSFM Reference Atlas of the Adult Mouse Brains

**Fig. 2:**
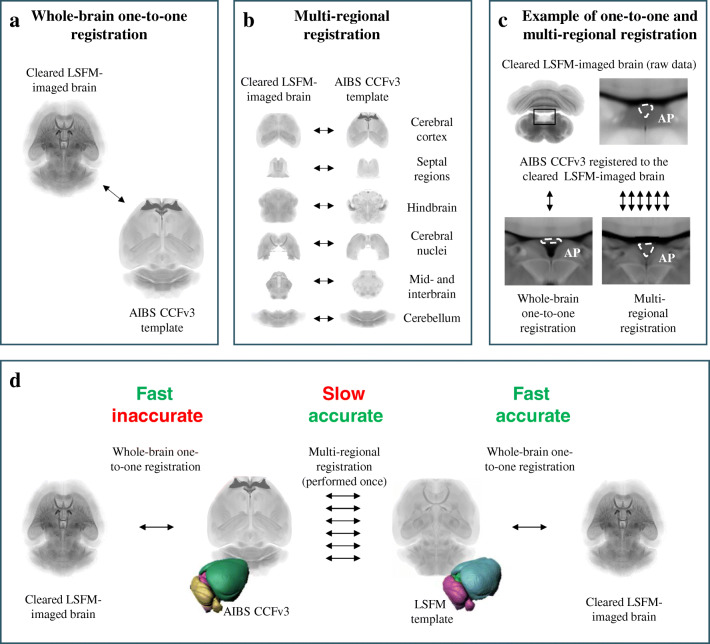
**Techniques for registering LSFM-imaged samples with the AIBS CCFv3.** a) Illustration of one-to-one registration between a cleared LSFM-imaged sample and the AIBS CCFv3 template. b) Illustration of multi-regional registration between a cleared LSFM-imaged sample and the AIBS CCFv3 template, where the brain volumes have been divided into six larger brain areas that are mapped individually. c) Example of the registration quality in area postrema (AP) using either one-to-one or multi-regional registration. d) Illustration of the registration flow described in this manuscript. Using one-to-one registration for aligning cleared LSFM-imaged samples with the AIBS CCFv3 is fast but inaccurate in some brain regions like the AP. Multi-regional registration for aligning cleared LSFM-imaged samples with the AIBS CCFv3 template provides better accuracy but is relative slow compared to the one-to-one registration. By generating a template from cleared LSFM-imaged brains and registering the AIBS CCFv3 with it once using multi-regional registration approach we ensure good alignment accuracy between the two templates. Subsequent registrations of cleared LSFM-imaged brains with the LSFM template can then be done directly using fast one-to-one registrations. This way it is possible to achieve both fast as well as accurate registration of cleared LSFM-imaged brains. Regardless of computer performance the speed of analysis improved by a factor of six compared to the multiregional registration

The standard way of aligning a LSFM scanned mouse brain with the AIBS CCFv3 is to perform a single cross-modality registration of the full brains by computing a global affine and local B-spline transformation in a one-to-one manner (Fig. 2a). However, an alternative strategy is to perform multiple registrations, where each of the major brain structures is aligned individually (Fig. 2b). By comparing the two approaches we observed that multiple registrations yield higher quality registrations in some parts of the brain, e.g. the area postrema (Fig. 2c). However, aligning LSFM-imaged brains using multiple registrations is time-consuming and require both initial segmentation of the larger brain structures and manual validation for each brain which is not compatible with high-throughput analysis. Our solution to this dilemma was to build an LSFM-based reference atlas by aligning the AIBS CCFv3 to the LSFM-based mouse brain template through multi-regional registrations. The present LSFM-based mouse brain reference atlas can be used to analyze individual LSFM-imaged samples directly by fast one-to-one registrations or for improved alignment to the AIBS CCFv3 space if needed (Fig. 2d). Regardless of computer performance we found that direct alignment to the LSFM atlas improved the registration speed for each brain sample volume by a factor of six.

An LSFM-based mouse brain reference atlas containing an average anatomy template with corresponding brain region annotations was created. The mouse brain template was generated from 139 3D autofluorescence-scanned brain volumes by an iterative multi-resolution image registration algorithm (Fig. 1a). Post-processing of the template involved refinement of the axial symmetry to obtain a midline symmetric atlas viewed from the coronal and horizontal orientation. The axial resolution of the mouse brain template is 20 μm. Brain region annotations for the LSFM template were imported from the AIBS CCFv3 by image registration (Fig. 1b). The annotations were imported as six separate pieces with manual corrections to mitigate the challenge of cross-modality registration. The final atlas contains 666 brain region segmentations with anatomical nomenclature corresponding to the AIBS CCFv3 (hierarchy tree of the atlas ontology in Online Resource 2) (Dong 2008).

### Improved Registration of LSFM-Imaged Mouse Brains

**Fig. 3:**
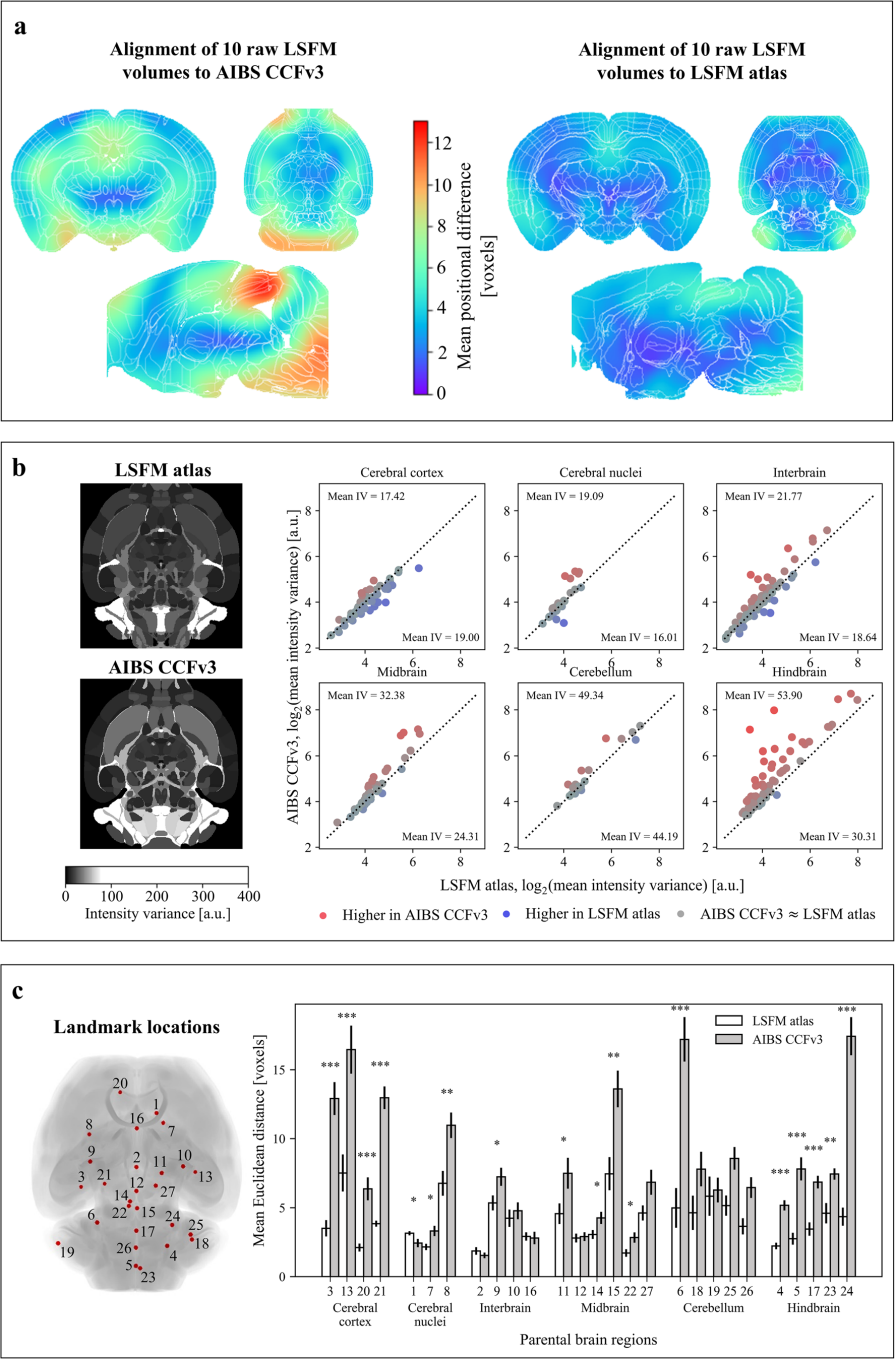
**Improved registration of LSFM-acquired brain volumes using the LSFM atlas.** a) Heatmaps illustrate the average magnitude of the deformation resulting from the registration of ten random raw LSFM brain volumes to the AIBS CCFv3 and to the LSFM atlas. b) Registration using the LSFM mouse brain atlas enables improved alignment between individual brains. Intensity variance, a measure for registration performance, was calculated per brain region for the ten random brain volumes aligned to the LSFM atlas and for the same ten brain volumes aligned to the AIBS CCFv3. Highest intensity variance was detected in both cases in ventricular and hindbrain regions (example sections, left). Statistical analysis of the intensity variance was performed using two-tailed Welch’s t-test and the resulting significant regions are visualized in the scatter plot (right) along with the mean intensity variance per major brain region for both atlases (denoted as mean IV). The results indicate that the difference in intensity variance values was small for cortical areas. However, majority of brain regions in cerebral nuclei, interbrain, midbrain, cerebellum and hindbrain exhibited higher intensity variance when the AIBS CCFv3 was used for registration compared to when the LSFM atlas was used for registration. c) Registration of the ten brain volumes was further evaluated using 27 landmarks distributed over the whole brain (overview of the landmark positions, left). The landmarks were divided between the six major brain areas in both atlases as well as in the ten brain volumes. Distances between the corresponding landmarks in the individual brains and the atlas templates were calculated after registering the ten brain volumes to the LSFM atlas and the AIBS CCFv3 (bar plot, right). For most landmarks, the calculated distances are lower when the LSFM atlas is used as template. Significant differences in distances between the two atlases was consistently observed in cerebral cortex and hindbrain. Two-tailed Welch’s t-test was applied for determining statistical significance in landmark distances between the atlases: ∗ for 0.01 ≤ *p* < 0.05, ∗∗ for 0.001 ≤ *p* < 0.01 and ∗∗∗ for *p* < 0.001

To validate that the LSFM reference atlas improved alignment of LSFM-acquired brain volumes, we tested alignment of ten raw LSFM-imaged mouse brain volumes and compared the results to alignment with the AIBS CCFv3 using identical registration procedures. By computing the amount of deformation needed to register each brain into the two atlases, we evaluated the voxel-wise magnitude of displacement necessary to convert the individual brain volumes to either of the atlas template (Fig. 3a). As expected, the LSFM-imaged brain volumes are less deformed when aligned with the generated LSFM atlas compared to alignment with the AIBS CCFv3. We found deformations ranging up to 13 voxels with the AIBS CCFv3 compared to deformations ranging up to 8 voxels with the LSFM atlas. Furthermore, the volume of the area affected by the deformation is smaller for the brains aligned to the LSFM atlas compared to the brains aligned to the AIBS CCFv3. The results show that deformations are most pronounced in the midbrain and hindbrain (Fig. 3a) and most likely the reflect they morphological changes inflicted by tissue processing and clearing.

As the magnitude of the deformation is only an indicative measure by which the registration quality cannot be fully assessed, we further investigated the alignment quality using a standardized metric called intensity variance developed by the Non-Rigid Image Registration Evaluation Project (NIREP) (Christensen et al. 2006). Intensity variance quantifies how much the signal intensity differs per voxel between the set of registered brain volumes and hence, estimates the amount of noise in the data set. We therefore computed the intensity variance for all brain regions using ten LSFM-imaged mouse brains registered to both the LSFM atlas and the AIBS CCFv3 (Fig. 3b). The mean intensity variance determined for the six major brain volumes registered to the AIBS CCFv3 was 17.42 for cerebral cortex, 19.09 for cerebral nuclei, 21.77 for interbrain, 32.38 midbrain, 49.34 for cerebellum and 53.90 for hindbrain. In contrast, the mean intensity variance computed for volumes registered to the LSFM atlas was 19.00 for cerebral cortex, 16.01 for cerebral nuclei, 18.64 for interbrain, 24.13 for midbrain, 44.19 for cerebellum, 30.31 for hindbrain. To analyse these findings in more detail, the intensity variance for all sub-regions within the six major brain regions, were plotted in scatter plots with AIBS CCFv3 values on the y-axis and LSFM atlas values on the x-axis. As for the deformation (Fig. 3a), the most substantial differences in intensity variance were observed in the midbrain and hindbrain. The improvement of the registration accuracy using the LSFM atlas was particularly notable for hindbrain due to significantly lower intensity variance for majority of the sub-regions when LSFM atlas was used for registration.

To further compare the registration quality between the two atlases, 27 landmarks were identified in both the LSFM and the AIBS CCFv3 templates, as well as in the same ten individual brain volumes which were previously used for registration evaluation (Fig. 3c; an atlas template containing the 27 landmarks together with the intensity variance map is available at GitHub and the atlas coordinates for each landmark can be found in the Online Resource 5). For the placement of each landmark several factors were considered. The landmarks should be: 1) easily recognizable in both the AIBS CCFv3 and LSFM templates; 2) distributed brain-wide such that several landmarks were located in cerebral cortex, cerebral nuclei, interbrain, midbrain, hindbrain and cerebellum; 3) distributed along the midline as well as in more lateral parts of the brain; 4) placed in regions with increased local intensity variance, if possible (Fig. 3b). Following the registration of the individual brains to the LSFM atlas and the AIBS CCFv3, the Euclidean distance between the registered and atlas landmarks was calculated. Although this approach also reflects the inherent variation that occurs when placing landmarks, it consistently showed more accurate registration when the LSFM atlas was used as a template.

### Accurate c-Fos Quantification in LSFM-Imaged Mouse Brains

**Fig. 4:**
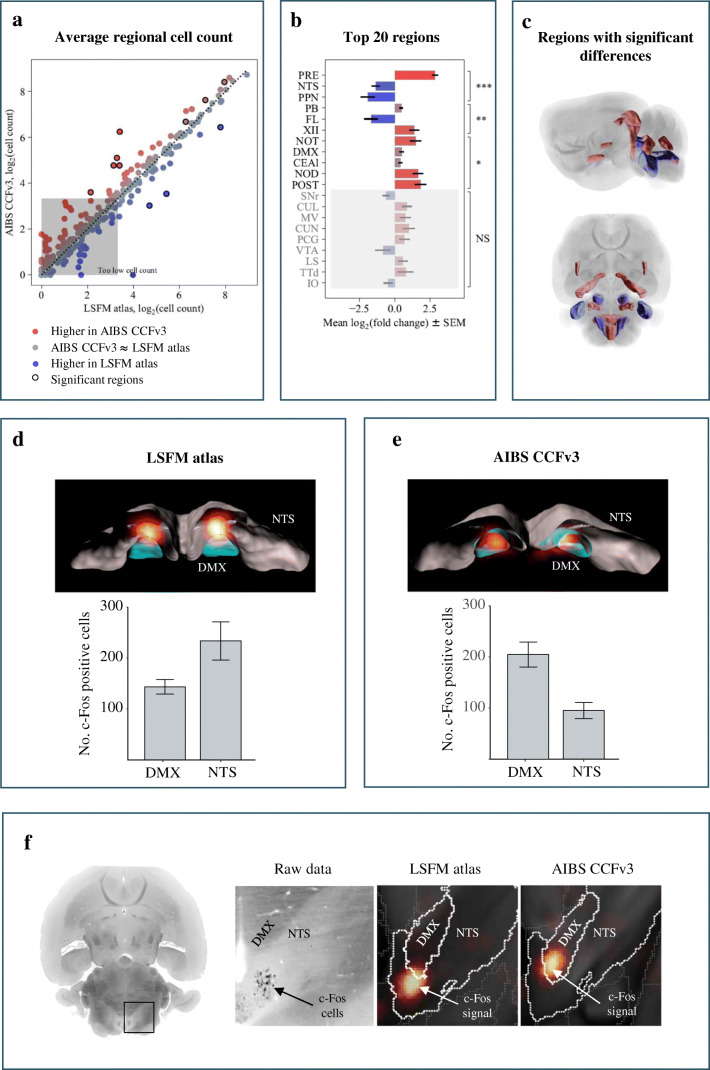
**Choice of brain atlas influences the number of c-Fos positive cells per brain region.** Comparison of number of c-Fos positive cells in response to semaglutide treatment using the LSFM atlas and the AIBS CCFv3. a) Average number of detected c-Fos expressing cells in every brain region after registration to either the AIBS CCFv3 or the LSFM atlas. Regions in which the c-Fos positive cells are differentially quantified are highlighted by a circle surrounding the data points. An average cell count per group below ten is considered too low to judge. b) The bar chart lists the brain regions and the corresponding mean log_2_ fold changes of quantified c-Fos positive cells in these regions according to the *p* value. Blue = higher with LSFM atlas, red = higher with CCFv3. NS stands for not significant, ∗ for 0.01 ≤ p < 0.05, ∗∗ for 0.001 ≤ p < 0.01 and ∗∗∗ for *p* < 0.001. c) Horizontally and sagittally depicted brain volumes highlight the regions in which the c-Fos cells were differentially quantified while using the LSFM atlas and the AIBS CCFv3 for the analysis (same colour code as in b and c). See Online Resource 2 for full names of the brain regions. d-e) Comparison of total number of c-Fos positive cells quantified in 3D-volumes of the nucleus of the solitary tract (NTS) and the dorsal motor nucleus of the vagus nerve (DMX) using the LSFM atlas and the AIBS CCFv3. DMX (blue) and NTS (grey) volumes of both atlases in which the signal (glow colormap) was quantified is visualized in 3D renderings. d) Quantification of c-Fos positive cells following registration of the LSFM atlas to the LSFM-acquired brain volumes showed that in average 234 ± 38 c-Fos positive cells were found in the NTS and 144 ± 14 in the DMX. e) Quantification of c-Fos positive cells following registration of the AIBS CCFv3 to the LSFM-acquired brain volumes. Here the majority of the signal is found in the DMX. Quantification revealed that on average 95 ± 16 c-Fos positive cells are counted in the NTS and 205 ± 25 in the DMX. f) Comparing the raw data to the data in alignment with atlases. DMX has a dense dark appearance compared to NTS

For evaluating the performance of the LSFM atlas to assign c-Fos positive cells to anatomical brain regions, we conducted a separate experiment where we mapped the brains from semaglutide-dosed lean mice onto the LSFM and AIBS CCFv3 atlas, respectively, and compared the distribution and number of c-Fos positive cells counted using each atlas (Fig. 4a). The two atlases showed highly overlapping results in the majority of brain regions. However, 11 regions showed significant differences in the number of c-Fos positive cells when comparing data analyzed with the two atlases (Fig. 4b-c). Hence, to determine how registration accuracy impacts the localization of c-Fos positive cells, we compared c-Fos signatures in the hindbrain regions, i.e. the nucleus of the solitary tract (NTS) and the dorsal motor nucleus of the vagus nerve (DMX). According to the LSFM atlas, most c-Fos positive cells were localized to the NTS (234 ± 38 cells) compared to the DMX (144 ± 14 cells) (Fig. 4d). In contrast, the AIBS CCFv3 revealed an opposite pattern (NTS, 95 ± 16 cells; DMX, 205 ± 25 cells) (Fig. 4e). To clarify which atlas is more accurate in the signal localization, we compared the raw microscope images to heatmaps representing c-Fos signal density using either atlas (Fig. 4f). The autofluorescence intensity of NTS is brighter than the intensity of surrounding tissue making it easy to delineate and shows that the raw c-Fos signal is indeed localized in the NTS, thus validating the LSFM atlas mapping. Signal localization accuracy of the LSFM atlas was also assessed for the other nine brain regions with conflicting c-Fos data (data not shown). While improved c-Fos signal localization by the LSFM atlas was confirmed for additional five regions (hypoglossal nucleus (XII), presubiculum (PRE), nodulus (NOD), nucleus of the optic tract (NOT) and postsubiculum (POST)). The AIBS CCFv3 performed better in one region, flocculus (FL), while three regions (lateral part of the central amygdalar nucleus (CEAl), parabrachial nucleus (PB) and pedunculopontine nucleus (PPN)), could not be properly evaluated because the ground truth could not be identified due to dispersed c-Fos signal.

### C-Fos Detection in a Pharmacological Study

**Fig. 5:**
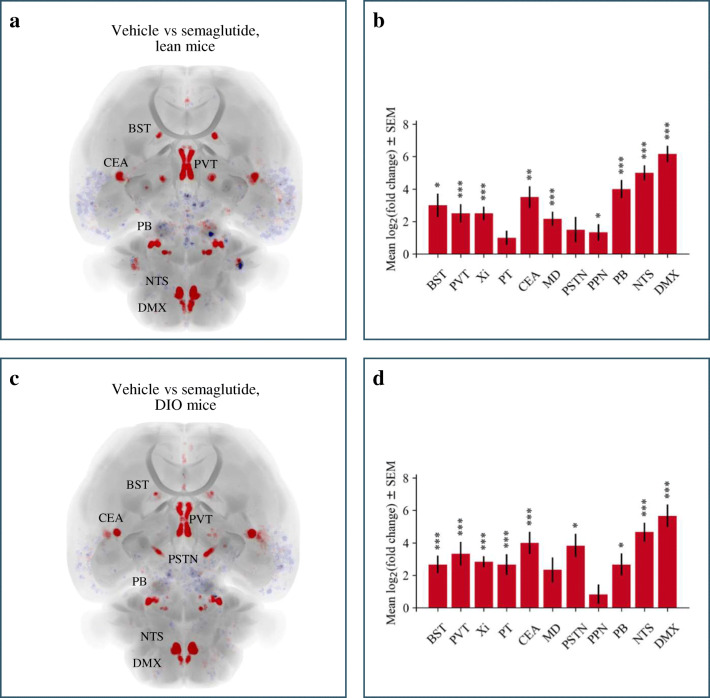
**Differentially regulated c-Fos expression in response to semaglutide administration.** Up (red) and down (blue) regulation of c-Fos expression in a) semaglutide treated lean mice in comparison to vehicle treated lean mice and c) in semaglutide treated DIO mice in comparison to vehicle treated DIO mice. Differentially regulated brain regions in response to semaglutide administration in comparison to vehicle treatment and corresponding mean log_2_ fold changes of c-Fos positive cells in these regions in b) lean and d) DIO mice. ∗ stands for 0.01 ≤ p < 0.05, ∗ ∗ for 0.001 ≤ p < 0.01 and ∗ ∗ ∗ for p < 0.001. *P*-values were adjusted for multiple comparisons using the false discovery rate. See Online Resource 2 for full names of the brain regions

To exemplify the use of the LSFM atlas we performed a study with the aim of quantifying c-Fos expression in mice dosed with the GLP-1 receptor agonist semaglutide. Semaglutide and vehicle was administered peripherally to lean and DIO mice, and the c-Fos expression was evaluated 4 h post-dosing (Fig. 5). When examining the raw LSFM volumes of DIO mice we observed increased autofluorescence in both the specific and the autofluorescence channel, which could potentially lead to false positive c-Fos signals (Supplementary Figs. 1 and 2). Increased autofluorescence in DIO mice was present throughout the brain, but strongest in the cerebellum (Supplementary Fig. 2). Since the increased tissue fluorescence was apparent in both channels, but true positive c-Fos signal was only present in the specific channel, the autofluorescence channel was applied for correction in whole-brain mounts in both lean and DIO mice (Supplementary Fig. 1), resulting in significantly improved signal-to-noise ratio specifically in DIO mice (Supplementary Fig. 2). To identify the differences between the semaglutide and the vehicle dosed mice, average signal heatmaps in semaglutide-treated lean and obese mice were subtracted voxel-wise from the corresponding vehicle control group (Fig. 5a, c) with statistical analyses on the raw c-Fos positive cell counts (Fig. 5b, d). Compared to vehicle controls, 9 brain regions were significantly regulated by semaglutide treatment in both lean and DIO mice. Semaglutide treated lean and DIO mice showed similar increased c-Fos expression in the bed nuclei of the stria terminalis (BST), paraventricular nucleus of the thalamus (PVT), xiphoid thalamic nucleus (Xi), central amygdalar nucleus (CEA), parabrachial nucleus (PB), nucleus of the solitary tract (NTS) and dorsal motor nucleus of the vagus nerve (DMX) compared to the vehicle treated controls. Additionally, semaglutide treated DIO mice exhibited increased c-Fos expression in the parataenial nucleus (PT) and parasubthalamic nucleus (PSTN), whereas semaglutide treated lean mice showed increased c-Fos expression in the pedunculopontine nucleus (PPN) and mediodorsal nucleus of thalamus (MD) compared to the respective vehicle treated controls.

## Discussion

We present here the generation of an LSFM-based mouse brain atlas. Compared to the AIBS CCFv3 (Allen Institute for Brain Science 2011, 2015, 2017; Kuan et al. 2015), the LSFM reference mouse brain atlas provides more accurate anatomical segmentation and quantitative detection of immunolabelled markers in iDISCO+ processed and LSFM-imaged mouse brains, exemplified by characterization of whole-brain c-Fos responses to semaglutide treatment in both lean and DIO mice.

To create an atlas template that is fully representative for average brain anatomy we developed the LSFM atlas based on the variational atlas algorithm previously described (Kovačević et al. 2005; Kuan et al. 2015; Umadevi Venkataraju et al. 2019). This algorithm avoids bias of the template towards the shape of a single chosen reference brain and accounts for morphological differences between the individual brains.

In comparison with the AIBS CCFv3, the created LSFM brain template resulted in registrations with lower amount of deformation, and the intensity variance as well as landmark distances showed improved alignment for LSFM-imaged samples. This is particularly relevant for tissue samples imaged with LSFM since the samples have been cleared and/or immunolabelled prior to scanning which affect brain morphology by shrinkage/expansion and de-lipidation (Kim et al. 2018; Wan et al. 2018). Furthermore, the contrast within anatomical structures in the brain that are important for subsequent image registration differs between the AIBS CCFv3 template and brain processed for LSFM. These issues have also been recognized by other researchers and a need for a dedicated atlas for cleared LSFM-imaged brains has previously been highlighted (Umadevi Venkataraju et al. 2019).

In this study annotations from the AIBS CCFv3 were mapped to the LSFM atlas template (Wang et al. 2020). However, as annotation volumes are continuously refined (Chon et al. 2019), these can also be aligned to the LSFM template. The process of mapping annotations from an existing atlas to the LSFM-template depends on cross-subject cross-modality registration (i.e. different brain, different microscope) which is difficult and often requires manual corrections. With the respect to mapping annotations from the AIBS CCFv3 to the LSFM-template, the main difficulty was related to morphology differences in the hindbrain and ventricular system. This was solved by stepwise mapping of the annotations for larger parts of the brain such as the hindbrain, together with manual corrections around the ventricular system. Now complete, the LSFM atlas provides the benefit of improved registration of other LSFM-samples together with detailed brain region annotations.

Our results demonstrate that c-Fos signal distribution in hindbrain regions is less accurately mapped using the AIBS CCFv3 compared to delineation of signals using our LSFM reference brain atlas. The large difference may be explained by the high amount of lipid-rich myelin fibers in this part of the brain (Smith 1973). As solvent-based tissue clearing removes lipids, this could explain the difficulty of mapping certain brain volumes to the AIBS CCFv3 which is based on non-cleared tissue samples. In addition to the NTS and DMX, we found improved signal localization using the LSFM atlas in five other brain areas. In four of these areas the improvement could be assigned to the detailed ventricular mask created for the LSFM atlas. Because the AIBS CCFv3 template depicts a narrower ventricular system compared to the LSFM atlas template, this may have resulted in incorrectly assigned c-Fos signal from the choroid plexus to nearby brain regions. In the FL, the AIBS CCFv3 performed better than the LSFM atlas. However, as the FL is often damaged or dislocated during dissection of the brain this may impact the subsequent mapping. In three brain regions we detected a significant difference in the mapping, but we were unable to determine which of the two atlases performed best because the c-Fos signal was too scattered.

In terms of c-Fos detection, DIO mice exhibited relatively high unspecific background signals as compared to lean controls, most likely attributed to lipid-associated autofluorescence. Lipid-containing residues of lysosomal digestion, lipofuscins, have also been reported to increase during aging and oxidative stress (Boellaard et al. 2004) leading to increased autofluorescence (Cho and Hwang 2011; Di Guardo 2015; Schnell et al. 1999). When comparing the c-Fos activity maps between lean and DIO mice we found that the response to semaglutide looked overall similar in both phenotypes with significant c-Fos activation in BST, PVT, Xi, CEA, PB, NTS and DMX. Semaglutide is a glucagon-like peptide-1 (GLP-1) analogue which has been shown to activate GLP-1 receptors in the hypothalamus and brainstem (Secher et al. 2014) and markedly stimulates c-Fos expression in mice (Kjaergaard et al. 2019; Salinas et al. 2018). The observed c-Fos expression pattern observed in this study fits well with these previous reports. Only slight differences were seen between lean and DIO as exemplified by only DIO mice showed significantly upregulated c-Fos expression in the PT and PSTN. It should be noted that lean mice demonstrated a similar c-Fos expression pattern in these regions which, however, did not attain statistical significance.

In this study a c-Fos was detected using a Cy5 labelled secondary antibody. Consequently we used the 560 nm to record the autofluorescence which is different from the mapping reported in the original ClearMap protocol (Renier et al. 2016). However, since the choice of fluorophores might vary from study to study, we tested how the choice of autofluorescence impacts the subsequent mapping (atlas-registered autofluorescence volumes can be found in Github). Although, we obtained the best registration using the 560 nm channel to record the autofluorescence, channels below 700 nm worked as well. When reaching the NIR spectrum the endogenous fluorescence become so weak it can no longer be used for registration.

The average brain generated in this study was created from 8 to 10 week old C57Bl/6 J male mice. Since factors such as age, sex and strain are known to affect brain size and anatomy, it is possible deviations from the average parameters may have a slight impact on the overall quality of registration and quantification. Indeed, we observed that obesity led to an unexpected increase in autofluorescence, presumably due to lipofuscin accumulation. In this case it did not impact on the registration, but it will always be important to consider the possibility that the choice of model may influence registration and quantification.

In conclusion, we developed a dedicated reference atlas allowing faster and more accurate mapping of iDISCO+ processed and LSFM-imaged whole mouse brains. In combination with an improved c-Fos detection algorithm, our pipeline enables for unbiased, automated and computationally efficient quantitative analysis of drug-induced c-Fos expression in the intact mouse brain. The LSFM atlas is highly applicable for fast and precise mapping of fluorescent markers in both the normal mouse brain and mouse models of CNS diseases as well for improved delineation of compound distribution in the CNS imaged by LSFM (Liebmann et al. 2016; Roostalu et al. 2019; Salinas et al. 2018; Secher et al. 2014).

## Information Sharing Statement

LSFM reference atlas files are freely accessible at https://github.com/Gubra-ApS. Quantitative c-Fos data for all brain regions is available as Online Resources 3 and 4. Source code used for generating the LSFM reference atlas along with the code for detecting and quantifying the number of c-Fos positive cells in LSFM mouse brain volumes is accessible at https://github.com/Gubra-ApS.

## Electronic supplementary material

ESM 1 (DOCX 36.1 kb)

Supplementary Figure 1 Improved detection of true c-Fos positive cells after removal of false positive c-Fos signal originating from increased tissue autofluorescence. a) Increased autofluorescence in DIO mice, apparent especially in hindbrain areas such as PB, can be recognized as c-Fos positive cells. b) Increased autofluorescence (red arrow) appear both in autofluorescence as well as in c-Fos specific channel. c) After channel alignment and background subtraction, false positive c-Fos signal is removed from the specific channel and d) true positive c-Fos cells (red cross) are quantified according to the ClearMap algorithm. (PNG 17.9 mb)

Supplementary Figure 2 Removal of the false positive c-Fos signal originating from increased tissue autofluorescence is an essential step for quantifying neuronal activity in DIO mice. This will be demonstrated in an example of heatmaps showing c-Fos response to semaglutide administration. a) ClearMap algorithm without the correction of false positive c-Fos signal performs well in lean mice. b) However, in old and obese mice brains there is an increased autofluorescence that can be detected as false c-Fos positive cells. c) Correction of false positive c-Fos signal does not have a strong impact on the signal fingerprint of young, lean mice, but d) will reduce notably the signal detected in DIO mice. (PNG 444 kb)

ESM 4 (XLSX 42.9 kb)

ESM 5 (XLSX 65.5 kb)

ESM 6 (XLSX 60 kb)

ESM 7 (CSV 435 bytes)
