# Supplementary material for: An Optimized Mouse Brain Atlas for Automated Mapping and Quantification of Neuronal Activity Using iDISCO+ and Light Sheet Fluorescence Microscopy
Source: Neuroinformatics. 2020 Oct 16;19(3):433–46. doi: 10.1007/s12021-020-09490-8 (PMC8233272; doi:10.1007/s12021-020-09490-8)
Supplement: Supplementary file 1 — (DOCX 36.1 kb) [file 12021_2020_9490_MOESM1_ESM.docx]

Online Resource 1 for An Optimized Mouse Brain Atlas for Automated Mapping and Quantification of Neuronal Activity Using iDISCO+ and Light Sheet Fluorescence Microscopy

Johanna Perens, Casper Gravesen Salinas, Jacob Lercke Skytte, Urmas Roostalu, Anders Bjorholm Dahl, Tim B. Dyrby, Pernille Barkholt, Niels Vrang, Jacob Jelsing and Jacob Hecksher-Sørensen ([jhs@gubra.dk](mailto:jhs@gubra.dk))

**Image processing for creating the LSFM reference atlas: full description and parameters**

An average LSFM mouse brain volume was created from 139 individual mouse brain autofluorescence datasets (Kovačević et al., 2005; Kuan et al., 2015; Umadevi Venkataraju et al., 2019). Pre-processing was initiated by down-sampling of the raw autofluorescence volumes to 20 µm isotropic resolution which was performed in axial plane using linear interpolating splines and in z-direction by local averaging of voxels. N3 method (Larsen et al., 2014; Van Leemput et al., 1999) was applied to down-sampled images for correcting the bias field caused by inhomogeneous fixation, clearing or illumination of the sample. Subsequently, contrast limited adaptive histogram equalization (CLAHE) was performed to achieve enhanced local signal contrast and higher visibility of different brain structures. CLAHE was carried out on 2D-images from the bias-field-corrected brain volumes sliced in three orthogonal planes (kernel size 1/3 of image height by 1/6 of 2D-image width, clipping limit 0.01, 255 histogram bins) and its results were then averaged together with the input bias-field-corrected volume. Then, a randomly chosen reference volume was rigidly aligned to the AIBS CCFv3 for realizing its axial symmetry. Pre-processing was finalized by matching the cumulative intensity histograms of the individual volumes with the cumulative intensity histogram of the reference volume.

To generate the average mouse brain template, a reference volume was selected for normalizing the individual brains in orientation, global size, gross shape and intensity. The algorithm was comprised of six iterative registration steps – one affine and five B-spline transformations. In the first step, all pre-processed mouse brain datasets were affinely registered to the pre-processed and CCFv3-oriented reference image according to a multi-resolution strategy and the resulting datasets were intensity averaged to generate an initial intermediate average brain. In the second step, the linearly aligned images underwent B-spline registration to the first intermediate average brain at the lowest resolution level. Subsequently, an updated intermediate average brain was produced from the non-linearly registered images. The remaining steps were analogous to the second step with the only difference being the resolution level. The highest resolution was reached at the last B-spline registration step.

Due to the limit in scanning depth in Z-dimension, which is about 6 mm for our LSFM setup, the acquired brain volumes missed about half a millimetre of the dorsal cortex. For producing a template with complete cortex, additional 15 autofluorescence volumes of cortices from the same animals were acquired, pre-processed and aligned through multi-resolution affine and B-spline transformations to the cortex reference volume. Then, the normalized cortex volumes were averaged, and the result was aligned through multi-resolution rigid, affine and B-spline transformations to the average mouse brain volume missing the top part of the cortex.   Subsequently, the final mouse brain template was produced by matching the intensities of both volumes and combing them by blending the overlapping areas with a sigmoidal function. Satisfying axial symmetry was achieved by dividing the template brain volume into three coronal slabs with equal thickness and manually rotating them into correct position. Subsequently, the three slabs were merged together using the sigmoidal blending function. The final template was created by mirroring one hemisphere to the opposite side and merging the hemispheres with the sigmoidal blending function for receiving a symmetric template brain.

The average LSFM mouse brain template was generated by registration steps which involved maximizing the mattes mutual information for fixed and moving image pairs. Multi-resolution strategy during affine registration was realized by increasing data complexity as well as transformation complexity throughout the 4 resolution levels. Data complexity was modified by smoothing and down-sampling (Gaussian pyramid scheme: σ_data_ = 8/2, 4/2, 2/2, 1/2 voxels) whereas transformation complexity was modified by changing the spacing of the control points on the grid (σ_transform_ = 100, 75, 50, 25 voxels). B-spline registrations were performed according to an uni-resolution strategy but by increasing the resolution with every B-spline registration step (σ_data_ = 8/2, 4/2, 2/2, 1.5/2, 1/2 voxels, σ_transform_ = 100, 75, 50, 35, 25 voxels. Parameters were optimized for every registration procedure to achieve the best possible spatial alignment for fixed and moving volume pairs.

Brain regions annotations were added to the LSFM template from the AIBS CCFv3. First, the mouse brain template of AIBS was registered to the LSFM template using multi-resolution affine and B-spline registration. Subsequently, the registered AIBS CCFv3 template and its segmentations were divided into six parental brain regions – cerebral cortex, cerebral nuclei, hindbrain, cerebellum, septal regions and interbrain together with midbrain. The parental regions were then separately registered to the corresponding areas of the LSFM template through affine and B-spline transformations and unified by filling in the border gap using distance map computation. This segmentation transfer approach led to misalignments in regions near to ventricular system, such as AP and SFO, which were therefore manually corrected. Further refinement of the segmentations was undertaken using Franklin and Paxinos mouse brain atlas for the ventral tegmental area (VTA) and the compact part of substantia nigra (SNc) as well as for the nucleus accumbens (ACB) which was divided into core and shell part. Segmentation refinements were performed with microscopy image analysis software Imaris™ version 2 (Oxford instruments, Abington, UK). Image processing was performed in Python and Elastix toolbox (Klein et al., 2010; Shamonin et al., 2014) was used to implement the registrations.

**REFERENCES**

Klein, S., Staring, M., Murphy, K., Viergever, M.A., Pluim, J.P.W., 2010. Elastix: A Toolbox for Intensity-Based Medical Image Registration. IEEE Trans. Med. Imaging 29, 196–205. https://doi.org/10.1109/TMI.2009.2035616

Kovačević, N., Henderson, J.T., Chan, E., Lifshitz, N., Bishop, J., Evans, A.C., Henkelman, R.M., Chen, X.J., 2005. A three-dimensional MRI atlas of the mouse brain with estimates of the average and variability. Cereb. Cortex 15, 639–645. https://doi.org/10.1093/cercor/bhh165

Kuan, L., Li, Y., Lau, C., Feng, D., Bernard, A., Sunkin, S.M., Zeng, H., Dang, C., Hawrylycz, M., Ng, L., 2015. Neuroinformatics of the Allen Mouse Brain Connectivity Atlas. Methods 73, 4–17. https://doi.org/10.1016/j.ymeth.2014.12.013

Larsen, C.T., Iglesias, J.E., Van Leemput, K., 2014. N3 Bias Field Correction Explained as a Bayesian Modeling Method. Bayesian Graph. Model. Biomed. Imaging. Lect. Notes Comput. Sci. 8677, 1–12. https://doi.org/10.1007/978-3-319-12289-2

Shamonin, D.P., Bron, E.E., Lelieveldt, B.P.F., Smits, M., Klein, S., Staring, M., 2014. Fast parallel image registration on CPU and GPU for diagnostic classification of Alzheimer’s disease. Front. Neuroinform. 7, 50. https://doi.org/10.3389/fninf.2013.00050

Umadevi Venkataraju, K.U., Gornet, J., Murugaiyan, G., Wu, Z., Osten, P., 2019. Development of brain templates for whole brain atlases. Prog. Biomed. Opt. Imaging - Proc. SPIE 10865, 1086511. https://doi.org/10.1117/12.2505295

Van Leemput, K., Maes, F., Vandermeulen, D., Suetens, P., 1999. Automated Model-Based Bias Field Correction of MR Images of the Brain. IEEE Trans. Med. Imaging 18, 885–896. https://doi.org/10.1109/42.811268
