# Supplementary figures and images for: An Optimized Mouse Brain Atlas for Automated Mapping and Quantification of Neuronal Activity Using iDISCO+ and Light Sheet Fluorescence Microscopy
Source: Neuroinformatics. 2020 Oct 16;19(3):433–46. doi: 10.1007/s12021-020-09490-8 (PMC8233272; doi:10.1007/s12021-020-09490-8)

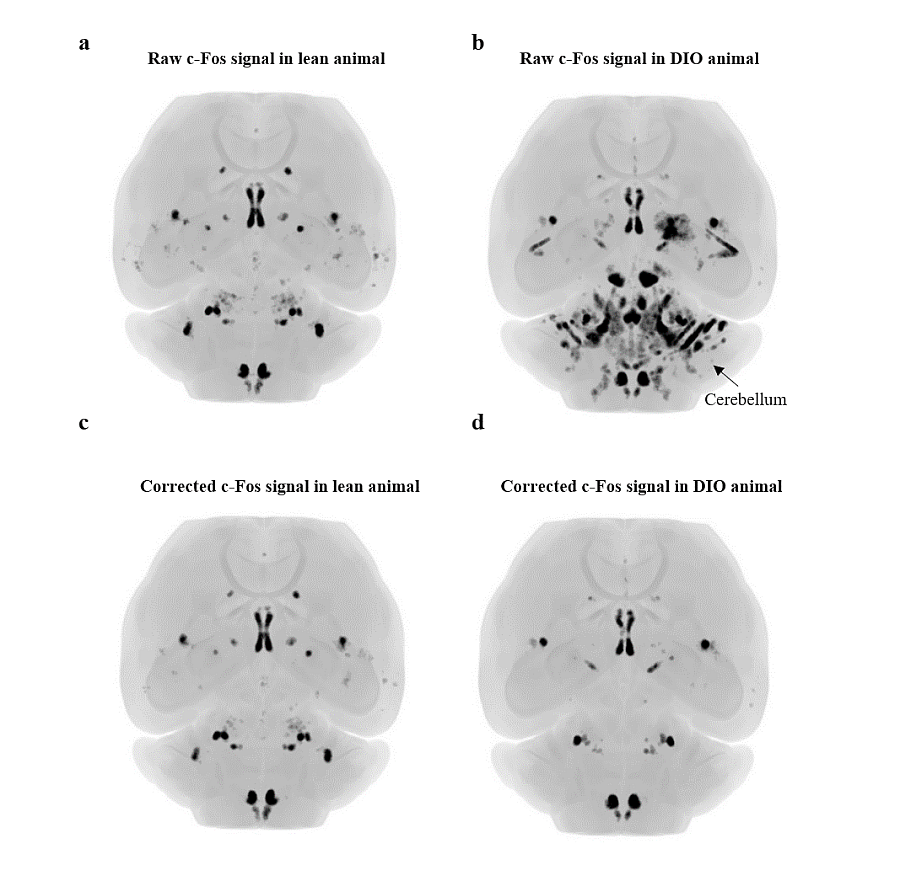

Supplement: Supplementary file 3 — Removal of the false positive c-Fos signal originating from increased tissue autofluorescence is an essential step for quantifying neuronal activity in DIO mice. This will be demonstrated in an example of heatmaps showing c-Fos response to semaglutide administration. a) ClearMap algorithm without the correction of false positive c-Fos signal performs well in lean mice. b) However, in old and obese mice brains there is an increased autofluorescence that can be detected as false c-Fos positive cells. c) Correction of false positive c-Fos signal does not have a strong impact on the signal fingerprint of young, lean mice, but d) will reduce notably the signal detected in DIO mice. (PNG 444 kb) [file 12021_2020_9490_MOESM3_ESM.png]
